# 2D vs. 3D Evaluation of Osteocyte Lacunae - Methodological Approaches, Recommended Parameters, and Challenges: A Narrative Review by the European Calcified Tissue Society (ECTS)

**DOI:** 10.1007/s11914-024-00877-z

**Published:** 2024-07-09

**Authors:** Annika vom Scheidt, Johannes Krug, Patricia Goggin, Astrid Diana Bakker, Björn Busse

**Affiliations:** 1 Division of Macroscopic and Clinical Anatomy, Gottfried Schatz Research Center, Medical University of Graz, Auenbruggerplatz 25, Graz, 8036 Austria; 2 Department of Osteology and Biomechanics, University Medical Center Hamburg-Eppendorf, Lottestr. 55a, 22529 Hamburg, Germany; 3 Interdisciplinary Competence Center for Interface Research, University Medical Center Hamburg-Eppendorf, Butenfeld 34, 22529 Hamburg, Germany; 4 Biomedical Imaging Unit, Laboratory and Pathology Block, University of Southampton, Southampton General Hospital, Tremona Road, Southampton, SO16 6YD UK; 5 Department of Oral Cell Biology, Academic Centre for Dentistry Amsterdam (ACTA), Amsterdam Movement Sciences, University of Amsterdam and Vrije Universiteit Amsterdam, Gustav Mahlerlaan, Amsterdam, 3004, 1081 LA The Netherlands

**Keywords:** Osteocyte, Lacuna, Lacunar volume, 3D Imaging, Microct, Nanoct, CLSM, Synchrotron

## Abstract

**Purpose of Review:**

Quantification of the morphology of osteocyte lacunae has become a powerful tool to investigate bone metabolism, pathologies and aging. This review will provide a brief overview of 2D and 3D imaging methods for the determination of lacunar shape, orientation, density, and volume. Deviations between 2D-based and 3D-based lacunar volume estimations are often not sufficiently addressed and may give rise to contradictory findings. Thus, the systematic error arising from 2D-based estimations of lacunar volume will be discussed, and an alternative calculation proposed. Further, standardized morphological parameters and best practices for sampling and segmentation are suggested.

**Recent Findings:**

We quantified the errors in reported estimation methods of lacunar volume based on 2D cross-sections, which increase with variations in lacunar orientation and histological cutting plane. The estimations of lacunar volume based on common practice in 2D imaging methods resulted in an underestimation of lacunar volume of up to 85% compared to actual lacunar volume in an artificial dataset. For a representative estimation of lacunar size and morphology based on 2D images, at least 400 lacunae should be assessed per sample.

**Supplementary Information:**

The online version contains supplementary material available at 10.1007/s11914-024-00877-z.

## Abstract (pa)

Osteocyte lacunae imaging methods have to be carefully selected regarding the aim of the project. If 3D imaging is not possible, 2D-based volume estimations should only be performed for samples with known lacunar orientation and elongation using a cutting plane aligned with the longest lacuna axis. The currently proposed recommendations for sampling and thresholding aim to guide researchers towards more reliable experimental results for elongated lacunae. Further research is required to determine the influence of method-inherent factors (contrast, resolution), segmentation, but also lacuna shape on lacunar volume estimation in order to come to best practices and comparable results within the bone community.

## Introduction

Although osteocytes and their lacunae have been of scientific interest since the 1950s [1], for decades their description was based on histological observation of thin sections, an inherently two-dimensional method. While these observations produced remarkable insight into the biological function of osteocytes and their intricate interaction with osteoblasts and osteoclasts [2], they were not able to capture the three-dimensional characteristics of osteocytes. It was impossible to assess their true volume and distribution within the whole bone, which are of high interest to bone researchers as lacunar morphological parameters are often altered with disease and represent potential biomarkers [3].

Fortunately, technical advances from such different fields as experimental physics, biochemistry, microscopy, and engineering lead to the emergence of multiple options to visualize both the osteocyte and the lacunocanalicular network in 3D over the last 20 years. Confocal laser scanning microscopy (CLSM), serial scanning electron microscopy (block-face or focused ion-beam (FIB)), and various types of tomography (electron, synchrotron- or desktop-based x-rays) can provide valuable information about lacunae, their dimensions, and distribution [4]. All mentioned methods have inherent advantages and limitations, offer specific resolutions and provide specific tissue contrasts.

Alongside the great opportunities emerging from three-dimensional analysis of osteocytes and their lacunae, controversies have arisen regarding complications in confirming 2D-based findings with 3D data. Previously established findings are being questioned as “2D measurements insufficiently predicted 3D outcomes from the same [..] lacunae” [5]. Furthermore, the parameters used to describe the osteocyte lacunar morphometry in 3D are inconsistent.

The aims of this manuscript are:

1. to offer a brief overview of the currently available methods for lacunae analysis,
2. to provide insight into the accuracy of 2D estimations of lacunar volume compared to the accuracy of true 3D volume measurements, by using simulated 2D and 3D measurements of idealized, artificial osteocyte lacunae, and through discussion of the influence of lacunar orientation and cutting planes, and
3. to recommend broadly applicable parameters for a uniform nomenclature for 3D lacunar morphometry and provide general considerations regarding sample size, resolution and thresholding.

### Prerequisites

The need for visualization and quantification of lacunae imposes several prerequisites on the imaging method:

1. The imaging method needs to be able to provide a contrast between the lacuna and the surrounding bone.
2. The method has to allow for the measurement of a representative volume (e.g. enable a sufficient penetration depth, capture a sufficient number of lacunae).
3. The resolution of the chosen method needs to be adequate to facilitate the detection of an expected effect size.

While researchers found many creative solutions to fulfill the first prerequisite that allows a clear distinction between lacunae and bone [6, 7], the two other prerequisites are more challenging. The reason for this is a lack of generalizable values for a representative volume size and a clearly established relationship between resolution and accuracy. Hence, this manuscript offers suggestions on how to determine a representative volume (or number of lacunae per sample) and a reasonable resolution for a planned experiment. It should be noted, that this paper focusses primarily on the analysis of lacunae. The analysis of canaliculi themselves, which house the dendritic cell processes of osteocytes projecting into the surrounding bone, requires a higher resolution than the analysis of lacunae. For a comprehensive evaluation of suitable methods for imaging and quantification of the lacunocanalicular network, the reader may refer to [8, 9].

## Methods for Analysis of Osteocyte Lacunae Morphology

Visualization of osteocyte lacunae is relevant for both fundamental and applied bone research because morphological parameters may serve as biomarkers to describe pathologies and monitor disease development [3]. Since all visualization methods rely on physical properties of the material, which depend on its composition, many methods will not allow a direct assessment of the osteocyte but rather an indirect assessment of their lacunae, as an enclosure in the surrounding bone matrix, which is why this manuscript focusses primarily on methods for visualization of lacunae. The visualization of osteocyte lacunae provides several challenges regardless of the method used. Firstly, the target of investigation is small (ellipsoid with approximate diameters of 19 × 9 × 5 μm [10]), thus the available resolution of the analysis method is of utmost importance. Secondly, the matrix surrounding osteocytes and their lacunae is calcified, requiring specialized processing methods with a potential of introducing artefacts [6]. Some techniques allow a direct assessment of the osteocyte and their lacunae, but just in demineralized bone or only in 2D. Consequently, the exact research question influences the method of choice. Thirdly, depending on the availability of samples, methodical aspects like sample destruction during measurement should be considered when choosing a method.

In the current literature several in-depth reviews of general 3D lacunae analysis are available [4, 6, 11, 12], while some authors have also reviewed specific methods [scanning electron microscopy (SEM): [7]; atomic force microscopy (AFM): [13]; synchrotron-based methods: [14, 15]; CLSM: [16]]. In the present manuscript, we primarily focus on methods for lacunae visualization. For a brief comparison, we will discuss their advantages and limitations in respect to the following aspects: ability to visualize in 2D or 3D, size of region or volume of interest (ROI/VOI), necessary/common preparation, current minimal resolution, applicability for canaliculi visualization, direct assessment of osteocyte or indirect via lacunae, method-inherent pros and cons, destructiveness of the method regarding the sample, and common artifacts. The following text will highlight the most relevant (dis)advantages. For an overview with all characteristics see Tables 1 and 2 for 2D and 3D methods, respectively.

### Visualization in 2D and Thin 3D Sections

While 3D methods are most useful for the visualization of lacunae, some commonly used 2D methods will be discussed here for the following reasons: (1) 2D methods have been and are still commonly used. (2) 2D methods can provide functional aspects through various staining techniques, e.g. labeling specific osteocyte metabolites or cell constituents. Thus, understanding the advantages and weaknesses of 2D methods compared to 3D methods for quantitative analysis of lacunar morphology is necessary to compare existing and future literature containing 2D and 3D results. Here, we will briefly discuss the pros and cons of conventional light microscopy, confocal laser scanning microscopy, scanning electron microscopy (+ acid etching), quantitative backscattered electron imaging (qBEI), transmission electron microscopy (TEM), and atomic force microscopy.

While for 2D methods, variations in lacunar morphometry along the axis perpendicular to the sample surface remain hidden, there may still be valid reasons for choosing specific 2D or semi-3D methods, depending on the research question. Reasons to choose these methods might be the wish to quantify the composition of bone matrix around lacunae without access to synchrotron imaging [17] or to correlate lacunar size with cell biological aspects. Why 2D-based estimations of lacunar morphology are problematic will be discussed using the example of lacunar volume in the Sect. Systematic Error with 2D-Based Estimates for 3D Objects.

#### Advantages of 2D Electron Microscopy Techniques (Acid etching-SEM, TEM, qBEI/BEI) and AFM

**Fig. 1:**
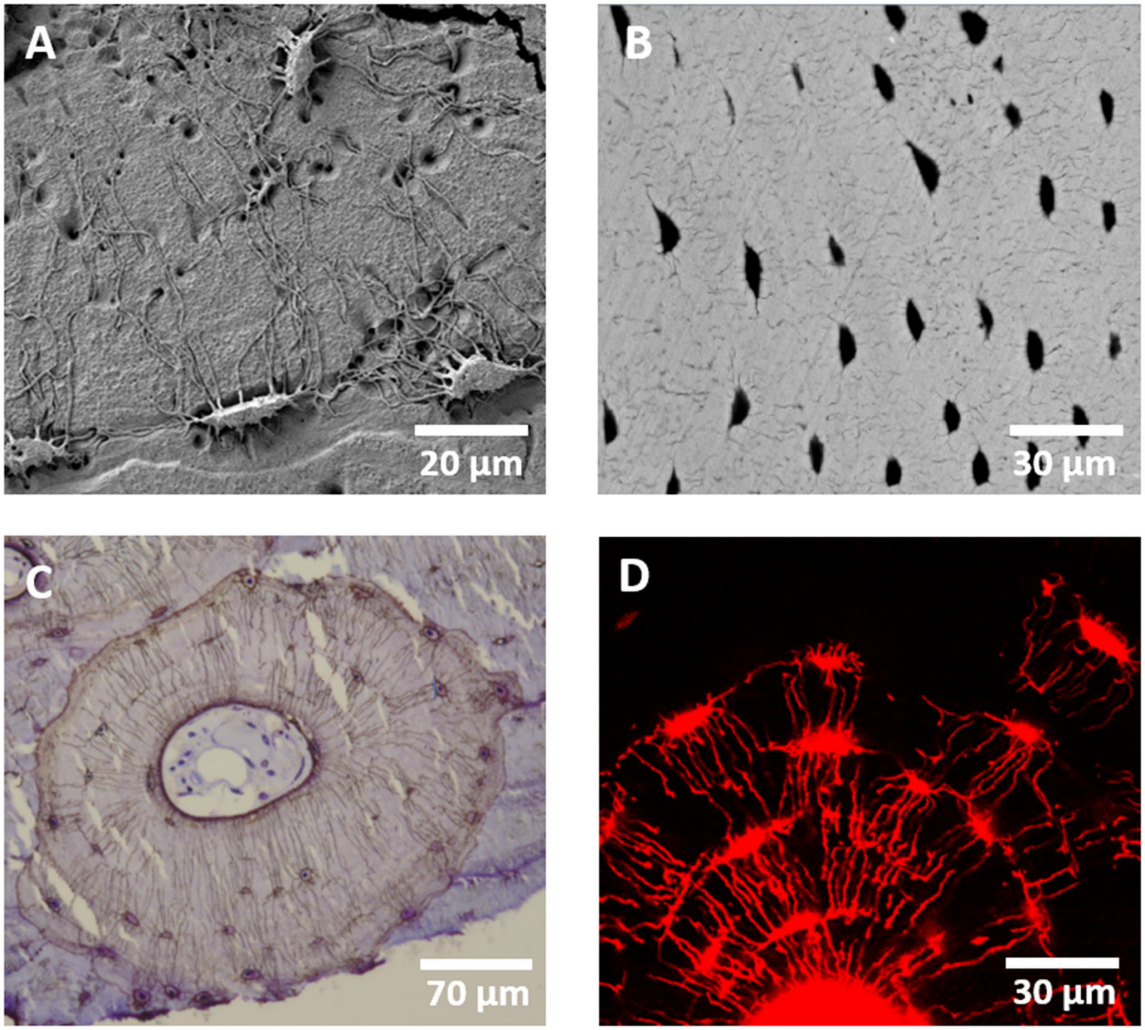
Exemplary images of lacunae and canaliculi visualized in 2D using (**A**) SEM on acid-etched bone, (**B**) qBEI (**C**) brightfield microscopy of a Ploton silver stained sample and D) confocal laser-scanning microscopy of a rhodamine stained sample

Electron microscopy offers different modes for lacuna and canaliculi visualization. Using secondary electron imaging after acid etching the surface of a PMMA-embedded bone sample allows to visualize the topography of the lacunocanalicular network in a bird’s eye view [18, 19] with a resolution in the nanometer range [20] and a comparatively simple preparation [19] (Fig. 1A). Alternatively, the organic component can be dissolved, resulting in voids where osteocytes and their canaliculi resided [21].

Further, transmission electron microscopy (= TEM) provides imaging of ultra-thin sections of bone with or without demineralization and optional staining [22, 23] at high-resolutions (0.2 nm) [11, 24]. Micro- and nanostructural features [25] as well as cellular and subcellular structures such as organelles or calcified nanospherites in micropetrosis [26] can be imaged and information about mineralization and crystallization can be retrieved [23, 27]. Depending on the detection mode, TEM can be used to visualize hard tissues (scattered electrons), soft tissues (phase contrast) [11] or more complex aspects, e.g. sample composition (high-angle annular dark field) or mineral crystallization (electron diffraction) [28].

Backscattered electron imaging (BEI) creates images with gray values based on atomic numbers and allows to analyze the mineralized matrix surrounding the lacunae and mineralized lacunae (micropetrosis) if present. The mineral dependent gray values not only provide high contrast between lacunae and the surrounding bone matrix but with additional calibration the mineral content can be quantified (quantitative backscattered electron imaging = qBEI) [29] and thus provide insight into mineralization processes (Fig. 1B).

Atomic force microscopy (AFM) theoretically offers up to atomic scale resolution [30] and can be used to create maps of lacunae recording their topography, mineral crystal size [31], and mechanical properties at the nanoscale [13, 32]. In the context of lacunae investigation, this method is predominantly used to investigate the bone surrounding lacunae and its mineralization [33–35] or to investigate objects within the lacunae, e.g. mineralized nanospherites [26], but it can also be employed to test the cytoskeleton of osteocytes [36]. Zhou and Du [30] provide a detailed overview of applications of AFM for bone research.

#### Limitations of 2D Electron Microscopy Techniques (Acid etching-SEM, TEM, qBEI/BEI) and AFM

All scanning electron microscopy-based techniques may be affected by artifacts stemming from crack development in vacuum, sample charging [37], and distortion effects (spatial distortion, drift distortion and scan line shifts) [38]. Correcting the latter is especially relevant if sample are mechanically tested and the deformation is evaluated using digital image correlation (DIC) [38]. Boyde [7] describes in detail best practices for the preparation of bone samples for SEM imaging.

Despite its straightforward sample preparation, SEM with acid etching has inherent limitations. The method is destructive since bone matrix is chemically removed during acid etching [19]. Consequently, the created image will display a mold of the lacunocanalicular network and the concentration of the acid and treatment duration influence the heterogeneity of the etching result [19]. Therefore, the accuracy of the network representation depends on the quality of the resin infiltration process, which may be affected by resin shrinking [11]. Due to the indirect imaging approach, samples with pathologies affected by osteocyte apoptosis or disruption of canaliculi might still show an intact lacunocanalicular network [19]. Further, the images represent a 2D projection of a 3D topography, which renders quantification of specific parameters difficult.

While TEM can provide very detailed insight into 2D properties of osteocytes and their lacunae, it requires complex sample preparation involving either FIB or ultra-thin sectioning, e.g. cryo-sectioning, as embedded bone samples have to be processed to reach a thickness of less than 100 nm [11]. Additionally, visualization of e.g. cell constituents may require optional staining [7].

Both variations of backscattered electron imaging (qBEI, BEI) can by themselves only produce 2D images. Fortunately, it is possible to combine them with focused ion beam milling or microtome cutting in the microscope chamber, allowing 3D visualizations with quantifiable mineral (calcium) content (cf. Sect. Advantages of Volume Electron Microscopy).

Due to its high resolution, the region of interest that can be measured in a realistic time frame is very limited for AFM. While AFM can be used on fresh, wet samples [13], often the surface of bone samples is processed through grinding or polishing, to ensure that height fluctuations are minimal. This might influence the recorded mechanical parameters [39]. Further, tip convolution can be a common source of artifacts [13] resulting in image distortions.

#### Advantages of Light, Confocal, Light Sheet, and Super-Resolution Light Microscopy

While conventional light microscopy offers 2D insights into lacunae morphology, confocal laser scanning microscopy (CLSM) and fluorescent light sheet microscopy allow 3D visualization of lacunae in thin samples but are limited by the penetration depth of bone to varying degrees.

All methods share the following advantages: (1) soft tissue contrast allowing the visualization of the osteocyte itself, (2) the possibility of mounting wet samples, (3) the option for functional imaging of subcellular and surrounding structures through histological staining, immunohistochemistry or fluorescent labeling.

Light microscopy with conventional staining methods has been used for bone histomorphometry for decades with plenty of detailed descriptions [1, 40] (Example of Ploton silver staining in Fig. 1C). It is limited by the diffraction limit of light (200 nm) [41]. Here, we focus on the advantages of confocal and light sheet microscopy in combination with fluorescent labeling. Although developed with other light sources [42], currently confocal microscopy most often makes use of lasers with specific wavelengths. These specific wavelengths can create auto-fluorescence in biological samples. As confocal microscopy is based on the detection of a point light source through a pinhole, which eliminates light outside of the focal plane [20, 43], blurring of the fluorescent signal is reduced in confocal light microscopy compared to conventional light microscopy [11].

While bone already possesses autofluorescent qualities [6], by combining specific wavelengths through filters or lasers with fluorescent dyes [44] it is possible to visualize osteocytes directly or indirectly and mark sub-cellular structures or other targets. Some fluorescent dyes stain the interstitial fluid and thereby allow indirect assessment of the osteocyte, common examples are: fluorescein isothiocyanate isomer I [45, 46], rhodamine based chromophore (Fig. 1D) [47], basic fuchsin [48]. Other fluorescent dyes or conjugated fluorescent dyes can directly stain the cell, its parts, or the surrounding matrix, e.g. dyes conjugated with phalloidin label the actin skeleton [49] whereas DAPI labels the nuclear chromatin/DNA [44]. A comprehensive list of fluorescent dyes, their applications, and the corresponding laser wavelengths can be found in Canette and Briandet [44].

In addition to osteocyte or lacuna visualization, CLSM can provide information about bone cell biology or bone growth when using tetracycline labelling [16, 50]. Some researchers have developed transgenic mouse models, which selectively express a membrane targeted-GFP (green fluorescent protein) variant in osteocytes, allowing optimized imaging of osteocytes without additional staining [51]. For more information on high-resolution fluorescence microscopy the reader may refer to Schermelleh, et al. [52].

Light sheet microscopy has become more popular in the last 10 years due to its ability to image large volumes in a short time with a high photon efficiency [4, 53]. Several studies highlight its potential for bone research by providing image volumes ranging from zebrafish craniofacial bones [53] to cleared whole porcine cochleae [54]. While the resolution of light sheet microscopy is worse compared to CLSM, light sheet microscopy allows sample sizes of up to 1 cm³ with clearing [4].

While super-resolution light microscopy methods like structured illumination microscopy (SIM), stochastic optical reconstruction microscopy (STORM), and total internal reflection fluorescence microscopy (TIRF) have become available and can supersede the resolution limit of conventional light microscopy, they have rarely been used to image osteocytes. SIM was used to image the lacunocanalicular network [55] and osteocyte processes in murine bone with a resolution of 90 nm [56].

#### Limitations of Light, Confocal Laser Scanning and Light Sheet Microscopy

Conventional light microscopy is limited to 2D imaging. For conventional light microscopy and CLSM, the histological sample preparation can introduce errors during cutting or microtome sectioning [20]. While both confocal light and confocal laser scanning microscopy are theoretically only restricted by the optical resolution limit and allow 3D visualization of thin samples, the depth of the volume of interest is limited due to the low photon penetrability of mineralized bone [20]. Depth-dependent artifacts arising from signal distortion and attenuation effects limit the attainable depth of the volume of interest in CLSM [6, 20]. While for mineralized bone samples the penetration depth of CLSM amounts to 10s of microns [57], for demineralized or cleared samples the penetration depth is much higher and can theoretically reach up to 150 μm [8]. Heveran et al. [5] recommend to image at least a depth of 60 μm to reduce sectioning effects of partially cut lacunae and to achieve stable values for 3D measures of osteocyte lacunar geometry. During evaluation, an inhomogeneity in lacunar contrast and bright bone marrow fluorescence can influence osteocyte (lacuna) morphology measurements specifically during thresholding [5].

Applications of light sheet microscopy for bone research have been limited by the need for translucency of the sample conflicting with the high density of bone. This issue can be overcome by tissue clearing methods optimized for bone allowing fluorescent 3D visualization of whole murine bones without sectioning using light sheet microscopy [54, 58, 59] and also visualization of osteocyte viability [60]. A drawback of clearing is the loss of information about the mineral content of the bone matrix due to demineralization during the process [59]. Furthermore, it is unclear how the cytoskeleton is influenced by the clearing process.

**Table 1:** Comparison of 2D osteocyte/lacuna imaging methods

Method	Light microscopy	SEM w/ acid etching	BEI/qBEI	TEM	AFM	Light sheet microscopy	Confocal laser scanning microscopy
2D/3D	2D	2D	2D	2D	2D	3D	2D/3D
ROI/VOI	large	large	large	small	intermediate	large but limited depth	large but limited depth
Min. resolution	200 nm	1 nm	> 1 nm	0.2 nm	5 nm	6.5 μm	200 × 200 × 300 nm
Common preparation	fixation, embedding, sectioning (4–25 μm)	fixation, embedding, acid etching, gold coating	fixation, embedding, polishing, carbon/gold coating	fixation, (staining), embedding, ultra-thin sectioning (100 nm), possibly decalcification	fixation, embedding or dried	fixation, clearing, commonly fluorescent dye	commonly fluorescent dye, fixation, (embedding), sectioning, decalcification for 3D
Visualization of canaliculi?	yes, with staining	yes	yes	yes	yes	no	yes, with staining
Direct/indirect assessment of osteocyte?	direct	indirect	indirect	direct	indirect	depending on dye	depending on dye
Pros	functional imaging, soft tissue contrast, wet mounting possible, simple, wide-spread availability	simple preparation	imaging of mineralized lacunae contrast based on composition	imaging of subcellular structures, resolution high enough for canaliculi, crystallization information in dark field mode	mechanical and topographical information about tissue	fast acquisition time, functional imaging through fluorescent labeling	functional imaging/labeling of subcellular structures using dyes, soft tissue contrast, wet mounting possible,
Cons	2D, limited analysis of lacunae morphology	2D representation of 3D network - quantification with care	calibration necessary, if mineral to be quantified (qBEI)	complicated preparation	calibration necessary to recognize tip wear	resolution not sufficient to assess osteocyte morphology, long preparation	anisotropic voxel size, blurred edges due to dye diffusion, overestimation of canaliculi
Destructive	no	yes	no	no	no	no	no
Artifacts	sectioning, dye inhomogeneity, cracks and folds from cutting	incomplete infiltration may prevent full imaging of canaliculi, potential shrinkage	insufficient coating, charging, electron beam damage, cracking in vacuum, shrinkage	sectioning, insuff. coating, charging, electron beam damage, cracking in vacuum, shrinkage	probe wear/ contamination, scanner artifacts (creep, nonlinearity)	sectioning, dye inhomogeneity	sectioning + dye inhomogeneity, (depth-dependent) artifacts through signal distortion & attenuation
Reference	[21, 41]	[7, 20]	[7, 29]	[24, 35]	[30]	[60]	[9, 16]

### Visualization in True 3D

A whole range of methods allows the visualization of osteocytes and/or their lacunae in 3D but all of these methods bring with them specific advantages and limitations. Here, we will discuss the most relevant dis(advantages), regarding size of ROI/VOI, preparation, minimal resolution, applicability for canaliculi analysis, direct/indirect assessment of osteocytes, sample destructiveness, and common artifacts. Tissue preparation with fixation, but also dehydration in alcohol and embedding, may lead to tissue shrinking [61], influencing results independent of the chosen imaging method. As with 2D methods, we primarily focus our attention to methods visualizing lacunae.

#### Advantages of Volume Electron Microscopy (FIB-SEM, Serial Block-Face SEM, Electron Tomography)

Volume electron microscopy is a fast-developing suite of methods, which provide insights into the 3D ultrastructure of cells and tissues. Both scanning electron microscopy (SEM) and transmission electron microscopy (TEM) (see Sect. Advantages of 2D Electron Microscopy Techniques) can also be employed for 3D imaging of lacunae. As the method-inherent sample penetration depth is low for bone, it is necessary to remove material in a destructive manner to make deeper planes accessible or to use very thin samples (< 100 nm thickness) and rotating scanning angles [62].

Volume SEM techniques include serial block face SEM (SBF-SEM), serial focused ion beam SEM (FIB-SEM) and array tomography (AT). All require heavy metal staining and resin embedding of tissue blocks. To enhance image contrast, the tissue may be stained using agents based on uranium, lead [63], osmium [64, 65], Lugol’s [66] or other chemicals [66, 67]. In SBF-SEM and FIB-SEM tissue blocks are repeatedly imaged and sectioned within the SEM chamber, with a diamond knife in a microtome and an ion beam respectively. In AT, serial sections are created with a diamond knife, collected on a substrate and imaged with an SEM, allowing repeated imaging. Each technique enables three-dimensional reconstruction of the volume.

FIB-SEM volumes can be acquired with a high spatial resolution in x-y (1 nm possible, 10–15 nm common [11]) and z (5 nm possible). Notably, isotropic voxels can be produced. Volumes imaged using FIB-SEM are typically smaller than SBF-SEM, and range from < 10 µm^3^ to 10^3^ µm^3^. As the imaging is perpendicular to the surface the depth is limited and thus volumes of this size are rare as their creation is very time-consuming and image quality is prone to suffer from material or beam instability, causing complications during post-processing [8]. Still, relatively large volumes have been successfully acquired at lower resolutions [68]. FIB-SEM provides the opportunity to image a sub-region of interest without discarding the rest of the blockface, allowing subsequent resampling.

SBF-SEM volumes are most often anisotropic, where x-y resolutions of approx. 5 nm are possible, but z resolution usually range between 40 and 100 nm. Volumes typically between 10^4^ and 10^6^ µm^3^ can be collected [69]. For a detailed description of advantages of lacuna imaging with SBF-SEM the reader may refer to Goggin, et al. [70].

While with FIB-SEM and SBF-SEM the surface layer of the sample is imaged, with electron tomography the sample is imaged in transmission in an ultra-high voltage electron microscope. Electron tomography allows for an investigation of the ultrastructure of bone [71]. The bone sample has to be very thin and is being rotated between acquiring transmission images equivalent to x-ray tomography. The image contrast is based either on elastically scattered electrons that pass through the sample or phase contrast [11, 72]. Similar to the other methods, a resolution of below 1 nm is theoretically achievable [72]. In combination with silver staining, cellular components can be visualized although the resolution is then dependent on the size of the silver particles (30–50 nm resolution) [73]. An emerging volume EM technology that has not yet been applied to bone is automated serial section TEM.

#### Limitations of Volume Electron Microscopy (FIB-SEM, Serial Block-Face SEM, Electron Tomography)

For FIB-SEM, the quality of milling and the resulting uniformity of material removal depends on the embedding medium and the homogeneity of the milled sample. High heterogeneity results in local variations of the milling rate and uneven milling planes [11], which can lead to unevenly spaced image slices in the reconstructed 3D stack. Ion beam shift and sample drift can impair image quality and have to be improved for bigger VOI [8]. Redeposition of milled material, charging of the sample during imaging and curtaining are further method inherent factors that may influence image quality [74, 75].

Many of the FIB-SEM specific limitations (beam shift, milling artifacts) can be circumvented by using SBF-SEM as no focused ion beam is needed and the sectioning is performed using a microtome. Unfortunately, due to the use of a microtome, microtome-specific artifacts like cracks or distortions can impair image quality. Additionally, the slicing process typically results in a non-isotropic 3D image, which can complicate quantitative analysis [70].

Since it relies on electron transmission through the sample, electron tomography is only possible for samples with a thickness of a few microns [6, 73].

While both FIB-SEM and serial block-face SEM are destructive methods, array tomography and electron tomography are not. Electron tomography offers a significantly smaller volume thickness (3–5 μm) and smaller total volume (~ 100 μm³) and is therefore predominantly useful to answer questions on the sub-cellular level [8]. All of these methods may incur charging and drift artifacts, as well as electron beam damage to the sample, making substantial amounts of post-processing necessary including slice alignment, histogram matching and stripe removal.

**Fig. 2:**
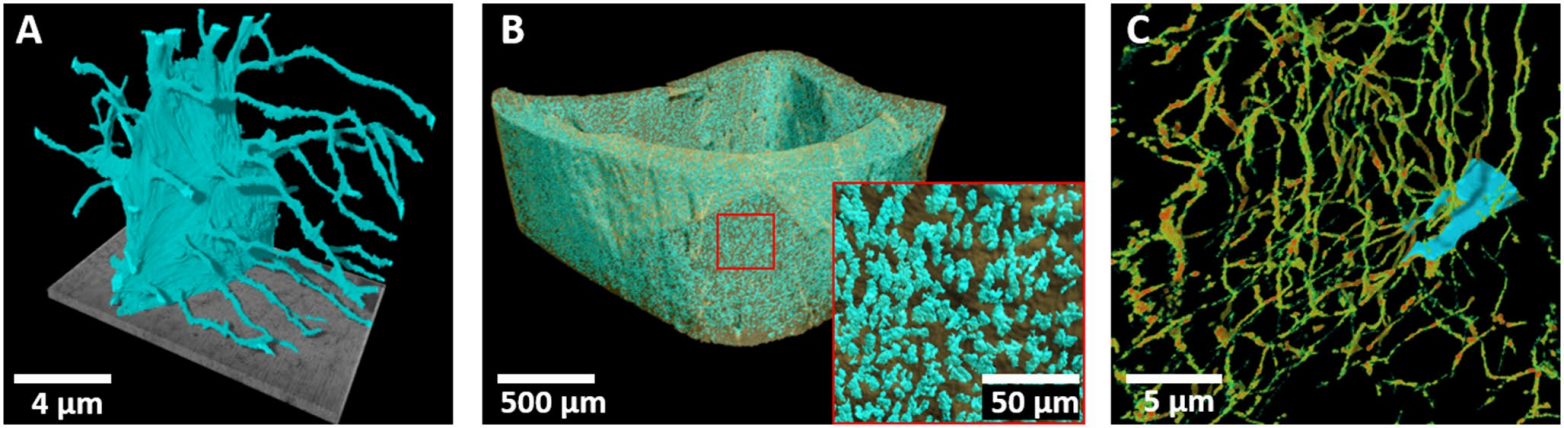
Examples for lacunae imaging in 3D using (**A**) FIB-SEM at 159 nm resolution, (**B**) large scale analysis using desktop-microCT at 700 nm resolution and (**C**) SR-phase-contrast CT at 45 nm resolution

As the VOIs in FIB-SEM typically only contain very few lacunae (Fig. 2a), this technique is not suitable for group comparisons of lacunae morphology or network analysis, but rather utilize the high resolution to investigate canaliculi, cell organelles or perilacunar/canalicular matrix mineralization [65, 67, 76, 77]. SBF-SEM offers the possibility to image larger VOIs with theoretically hundreds of lacunae as larger areas can be sectioned.

#### Advantages of X-ray-Based Tomography (microCT, nanoCT, X-ray Microscopy

In this section, we will discuss commercially available x-ray-based tomography instruments. Setups based on synchrotron radiation will be discussed in the next section. X-ray-based tomography allows to analyze osteocytes indirectly by visualizing their lacunae in 3D in a non-destructive manner and, depending on the instrument, offers a wide range of resolutions. MicroCT, nanoCT, and x-ray microscopy operate similarly to clinical CT scanners. In contrast to clinical CT scanners, for these methods, the sample is rotated and the x-ray source and detector are fixed. As the methodological basis is the same, there is no strict definition for a clear differentiation between microCT and nanoCT. Depending on the manufacturer, for an instrument with a resolution of ~ 0.5 μm either the term microCT or nanoCT may be used. Here, we will use the term microCT imaging for resolutions down to approximately 1 μm, and nanoCT imaging for instruments with a resolution of down to 150 nm [78]. Despite the fluid differentiation between these two instrument types, it is important to note that a higher resolution is always accompanied by a smaller imageable volume of interest. While common microCTs theoretically allow a sample size in the range of multiple centimeters, they cannot achieve a high-enough resolution to image osteocyte lacunae for samples of this size. Thus, sample thickness in transmission direction is restricted to a few millimeters [79, 80], which still allows to image thousands of lacunae (Fig. 2B). Hence, two of the greatest advantages of microCT imaging are the ability to acquire large, representative volumes of bone, and the assessment of spatial lacunar relations [81].

Although some x-ray microscopy instruments are also called nanoCTs, owing to their high resolution, we are discussing them separately, as they use a specific technique to achieve this high resolution. This specific technique uses zone plates [82], which act as lenses for x-rays and allow to circumvent the diffraction limit of light. Due to this technique, x-ray microscopy enables the user to scan at a much higher resolution than other nanoCT instruments (~ 30–50 nm vs. ~150 nm, respectively), although only samples with a size of tens of microns can be imaged at those magnifications (~ 15–65 μm), theoretically allowing the visualization of lacunae with canaliculi [83]. At lower resolutions, X-ray microscopes can still be used to visualize larger regions of interest, similar to microCT and nanoCT [84, 85].

For all three techniques, sample preparation is easy as no specific fixation or embedding is necessary. Samples can be scanned mounted in air or liquid, but it is of utmost importance to fix them in their position to avoid movement artifacts [86].

#### Limitations of X-ray-Based Tomography (microCT, nanoCT, X-ray Microscopy

Considering the limitations of sample sizes discussed above for x-ray-based tomography instruments, it is important to choose methods strictly based on the research question. If the additional visualization of canaliculi is important, depending on the resolution nanoCT and theoretically x-ray microscopy are appropriate.

As these methods are using x-rays for visualization, soft tissue is invisible without adequate staining solutions (e.g. Lugol’s, osmium), which can infiltrate the tissue and adhere to cell constituents to varying degrees, subsequently increasing attenuation and thereby contrast. Typical CT artifacts like beam hardening, ring artifacts, metal artifacts or artifacts due to movement/heat expansion are common but can be reduced with appropriate settings (x-ray filters, 360° scans, averaging of images, etc.) [87] and post-processing. Furthermore, the choice of an appropriate thresholding method is of utmost importance in microCT imaging, as partial volume effects can greatly affect the accuracy of image binarization and therefore lacunar volume assessment at lower resolutions [81].

#### Advantages of Synchrotron-Based Tomography (SR-phase-Contrast CT, SR-microCT/nanoCT)

The high-flux, coherent x-ray beam produced by synchrotron radiation (SR) facilities allows to acquire CT scans following the same principle as desktop microCT and nanoCT instruments to asses lacunae [88], but with much lower acquisition times and reduced beam hardening artifacts, allowing for the assessment of local spatial gradients of bone mineral density [14]. In contrast, for desktop microCT and nanoCT solutions, beam hardening hinders an exact direct assessment of local spatial gradients of volumetric bone mineral density [89]. Additionally, a variety of techniques utilize phase contrast in combination with phase retrieval algorithms to gain 3D reconstructions (e.g. ptychographic CT, SR-holotomography). As phase contrast is dependent on changes of refractive indices between materials, it generates sufficient contrast for soft tissue and cavities that are not achievable with absorption contrast [14]. SR-phase-contrast CT resolution is not limited by the wavelength of light, hence lacunar networks have been visualized with voxel sizes of around 30 nm [90], and ptychographic CT reaches a similar resolution [91, 92] (Fig. 2C). For comprehensive reviews of synchrotron-based tomography, the reader may refer to Obata, et al. [15] and Portier, et al. [14].

#### Limitations of Synchrotron-Based Tomography (SR-phase-Contrast CT, SR-microCT/nanoCT)

The main limitation of all synchrotron-based methods lies in the fact that access to synchrotron beamlines mostly has to be granted through a competitive application process and thus complicates project planning. In addition, absorption contrast SR-CT offers no soft tissue visibility and presents ring artifacts just as commercially available desktop microCTs or nanoCTs [15]. Further, the volume that can be imaged is limited at very high resolutions [90], although with a resolution of 0.76 μm samples of more than 3 mm length can be imaged using phase-contrast CT [14]. For very high resolutions, it might be necessary to use cryoconservation to limit radiation damage during ptychographic CT imaging [93].

**Table 2:** Comparison of 3D osteocyte/lacuna imaging methods

Method	Serial block-face SEM	Serial FIB-SEM	TEM-CT/ electron tomography	Phase-contrast SR-CT	Desktop microCT/nanoCT	X-ray microscopy CT	Absorption SR-µ/nanoCT
2D/3D	3D	3D	3D	3D	3D	3D	3D
ROI/VOI	small	very small	very small and limited depth	intermediate-very small	large-small	large-very small	large-small
Min. resolution	10 × 10 × 50 nm	< 5 nm	2 nm	30 nm	150 nm	50 nm	325 nm
Common preparation	fixation, heavy metal stain, embedding, gold coating	Fixation, heavy metal stain, embedding,	fixation, heavy metal stain, embedding, sectioning	fixation, embedding, (FIB/laser) sectioning	cutting	cutting, (FIB/laser) sectioning	dehydration, cutting,
Visualization of canaliculi?	yes	yes	yes	yes	no	potentially	yes
Direct/indirect assessment of osteocyte?	indirect direct with staining	indirect direct with staining	direct	indirect	indirect	indirect	indirect
Pros	large VOIs, resolution high enough for canaliculi	imaging of subcellular structures in 3D with staining, isotropic data	imaging of subcellular structures in 3D	quantitative assessment of density without calibration	simple sample preparation, large scale analysis	simple sample preparation, large range of resolutions	direct assessment mineral density, faster than desktop
Cons	artifacts, no soft tissue visibility without staining, non-isotropic data	no soft tissue visibility without staining, long acquisition time	complicated preparation (FIB milling)	synchrotron access, time intensive, possible radiation damage, long acquisition time at high resolutions	no soft tissue visibility without staining, long acquisition time, possible minor radiation damage	no soft tissue visibility without staining, long acquisition time, possible minor radiation damage	synchrotron access necessary, staining for soft tissue visibility, radiation damage possible
Destructive	yes	yes	no	no	no	no	no
Artifacts	insufficient conductivity, charging, electron beam damage, cracking, shrinkage	curtaining through redeposition, insufficient coating, charging, electron beam damage, cracking in vacuum	sectioning, no full 180° scan, ‘missing wedge’, shrinkage	instability of source, ring artifacts, movement/heat expansion, phase-contrast artifacts, potentially shrinkage	beam hardening, ring artifacts, movement/heat expansion, metal	beam hardening, ring artifacts, movement/heat expansion, metal	ring artifacts, movement/heat expansion, metal (limited), potentially shrinkage
References	[4, 70]	[8, 65]	[72, 73]	[14, 90]	[3, 78]	[83, 84]	[15, 88]

## Systematic Error with 2D-Based Estimates for 3D Objects

Before providing a reasonable sample size for 2D analysis of lacunae it has to be acknowledged that all 2D-based estimations of these three-dimensional objects (elongated ellipsoids with an axes ratio of approximately 4:2:1 [10]) include a systematic error. This holds true both for one-dimensional and three-dimensional parameters. In respect to one-dimensional parameters such as minimum or maximum diameter the error occurs due to cutting the lacunae in a random cross-section. When analyzing 2D cross-sections of lacunae, the maximum diameter of the cut cross-section of the lacunae (${D}_{\text{m}\text{a}\text{x}\_cut }$) will be used as an estimate, while the actual longest diameter of the lacuna ${D}_{\text{m}\text{a}\text{x}\_3D}$ is likely greater. Two possible causes may lead to misrepresentation of the dimensions of a lacuna in 2D: (1) The longest diameter of a lacuna, ${D}_{\text{m}\text{a}\text{x}\_3D}$, can only be measured if the cutting plane intersects the center of the lacunae along its longest axes. Every parallel cutting plane will show a smaller lacunar cross-section and underestimate the maximum lacunar diameter. (2) Rotation of the lacunae or tilted sectioning of a sample will likely exacerbate this issue. E.g. when cutting a lacuna perpendicular to its longest axis, the measured maximum diameter of the 2D representation, ${D}_{\text{m}\text{a}\text{x}\_cut}$, vastly underestimates the true diameter. These one-dimensional issues translate into the estimation of three-dimensional parameters such as lacunar volume. Consequently, here the underestimation is even more drastic through the cubic nature of volume calculations from scalars. A common calculation for a 2D-based estimation of lacunar volume ${Lc.V}_{pre}$ uses the following Eq. [1]:

1 $${Lc.V}_{pre}=\frac{\pi }{6}*{D}_{\text{m}\text{i}\text{n}\_cut}{*D}_{\text{m}\text{i}\text{n}\_cut}*{D}_{\text{m}\text{a}\text{x}\_cut}$$

with ${D}_{\text{m}\text{i}\text{n}\_cut}$ being the smallest diameter of the lacuna in the cutting plane and ${D}_{\text{m}\text{a}\text{x}\_cut}$ being the largest diameter. Using this equation, a previous study showed an underestimation of 42–66% for lacunae with a moderate 0-22.5° orientation angle and an underestimation of up to 34% for randomly oriented lacunae (using 0.33 μm resolution) [12]. These drastic underestimations occur, even though in the mentioned study the lacunae were only cut through their respective mid-sections, which resulted in the highest possible volume estimations, not reflecting real 2D assessments.

It has to be noted that the elongation of the lacunae and the resulting axes ratios influence the quality of the estimation. For lacunae with stronger elongation than the here chosen 4:2:1 ratio, the error in estimation of the lacunar volume should be amplified, while for less elongated, rounder lacunae the error in estimation is likely smaller. For the sake of this review we focus on elongated lacunae with a 4:2:1 ratio, but we caution readers to directly transfer the provided reasoning for analysis of lacunae with smaller elongation, specifically round lacunae.

### Realistic Estimation Errors in Relation to Lacunae Orientation, Cutting Plane and Resolution

**Fig. 3:**
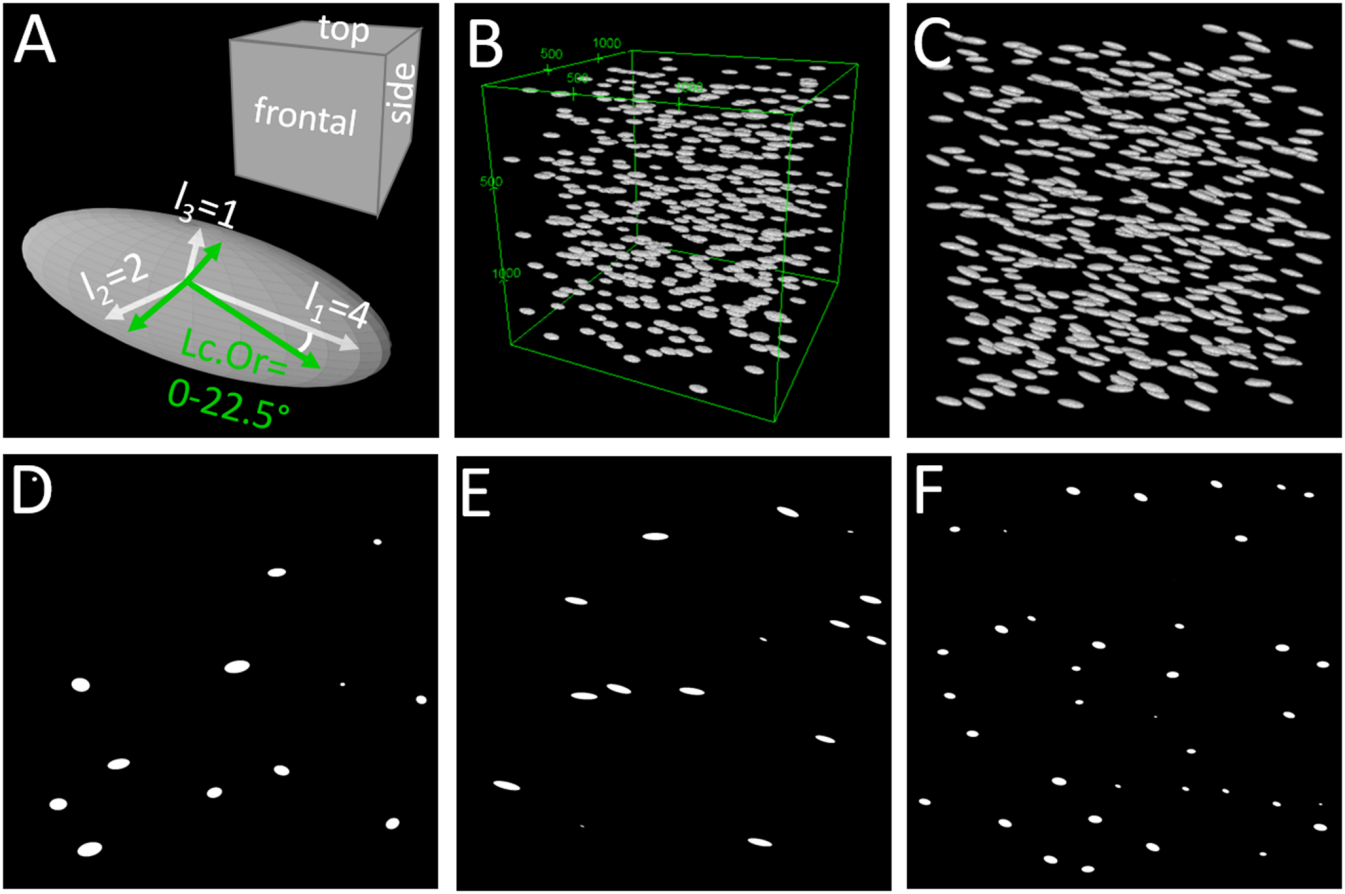
**A**: Naming convention for cutting planes and representative lacuna/ellipsoid. **B**: 3D representation of artificial lacunae dataset with idealized lacunar orientation (0°). **C**: 3D representation of artificial lacunae dataset with realistic lacunar orientation (0-22.5° angle, 0.2 μm resolution). **D**: Top cutting plane parallel to two longer axes of lacunae. **E**: Frontal cutting plane parallel to longest and shortest axis of lacuna. **F**: Side cutting plane parallel to two shorter axes of lacunae

To create an increased understanding of the errors inherent in 2D-based volume estimation, we created artificial 3D datasets representing a bone volume with 500 lacunae. To emulate the varying orientations of lacunae throughout the skeleton [47, 94] in different bone types due to their alignment with collagen, we created artificial bone volumes with different lacunar orientations (0°, 0-22.5°, random) at different resolutions (0.2–1.45 μm). Artificial bone volumes were created using custom code in Python 3.9 (Python Software Foundation) with implementation of the PyEllipsoid package by Andrei Shkarin. These 3D volumes were digitally cut into 2D slices from three planes to emulate 2D methods and evaluated with Fiji [95]. The cutting planes are parallel to the surfaces of the artificial bone volume (frontal, top, and side plane, cf. Figure 3 A&B). For lacunae with 0° orientation this means, the top plane is parallel to the long and the medium axis of the lacunae, the frontal plane is parallel to the long and short axis of the lacunae, and the side plane is parallel to the medium and short axis. Figure 3 C-F show an artificial bone volume with lacunar orientations between 0-22.5° and examples of the resulting 2D slides illustrating the vastly different appearance of lacunae depending on the cutting plane. For details the reader may refer to the supplementary methods section.

#### Influence of Cutting Plane

**Fig. 4:**
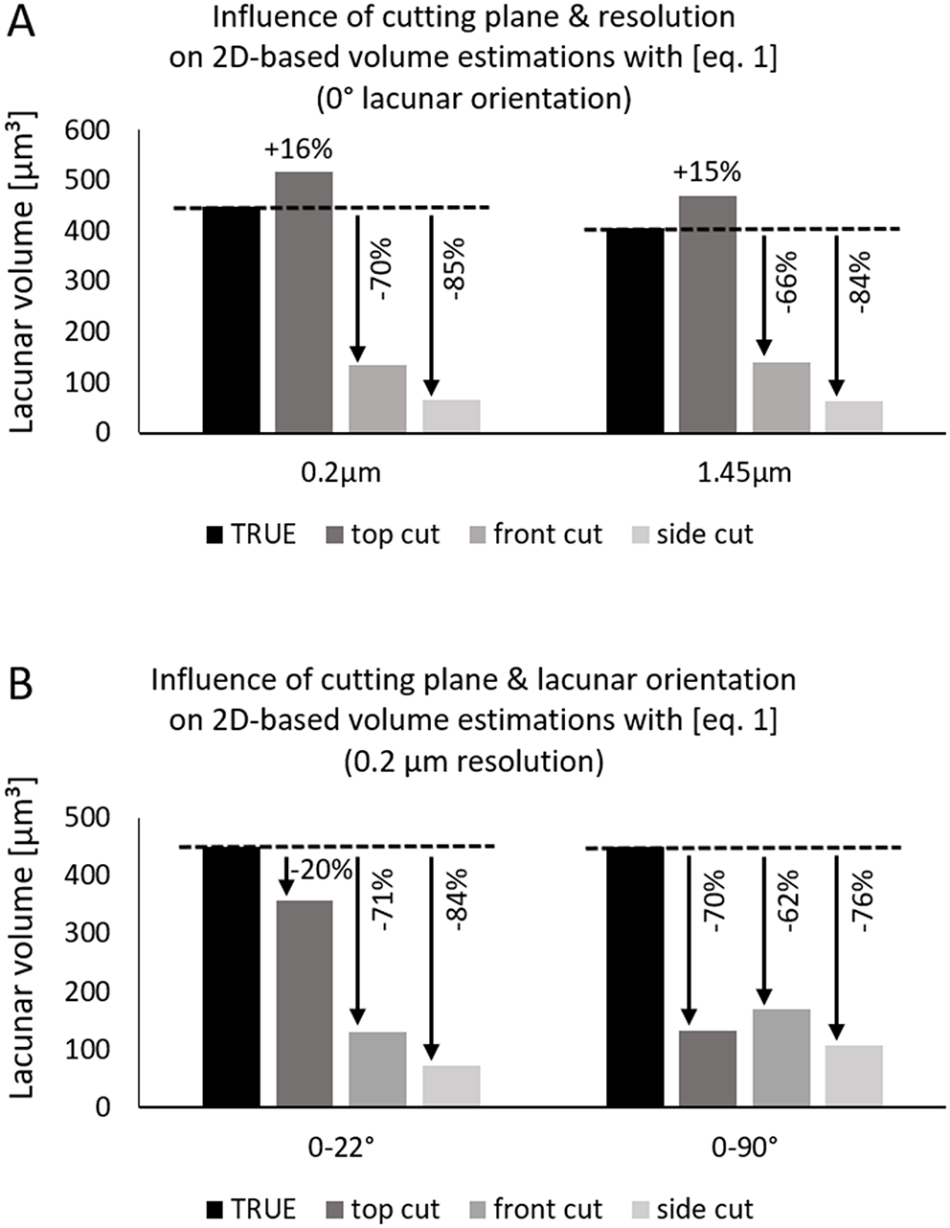
Exemplary over- and underestimation of lacunar volume using [Eq. 1] on cross-sections of an artificial lacunae dataset. **A**: Different cutting planes result in drastically different over- or underestimation for both tested resolutions (0.2 μm, 1.45 μm). **B**: For realistic lacunar orientations in lamellar bone (0–22°) or randomly oriented lacunae, lacunar volume is always underestimated

For lacunae perfectly aligned with the outer surfaces of the artificial volume (lacuna axes parallel (0°) to volume axes), we analyzed the influence of cutting planes. We created lacunae with approximate individual volumes of 406.5 μm³ for 1.5 μm resolution and 448 μm³ for 0.2 μm resolution with an aspect ratio of 4:2:1. Using [Eq. 1] and a resolution of 1.45 μm, we found an overestimation for the top cutting plane (parallel to the two longer axes of the lacunae) of 15.4% but an underestimation of 65.5% and 84.4% for frontal and side cutting planes (parallel to the longest and shorter axis or parallel to the two shorter axes). For a resolution of 0.2 μm, the values are very similar, reaching 15.6% overestimation and underestimations of 70.0% and 85.2% respectively, cf. Figure 4. This corresponds to the variation within bone samples, where values will vary slightly depending on the chosen volume of interest.

#### Influence of Lacunar Orientation

Assuming realistic lacunar orientation with angles between 0 and 22.5° for all axes (cf. Figure 3 C), lacunar volumes are always underestimated, independent of resolution: 19.6–20.2% underestimation for the top plane, 70.6–70.7% underestimation for the frontal plane and 84.1–85.1% underestimation for the side plane, for resolutions of 0.2 and 1.45 μm. Considering randomly oriented lacunae like in woven bone (simulated angles between 0–90°) results in an underestimation of 65–78% for 1.45 μm resolution and 62–76% for 0.2 μm resolution for all three planes. It should be noted that the exact values will vary slightly if the artificial volumes are recreated due to the randomized distribution of lacunae within the volume.

It has to be strongly emphasized that without prior knowledge about the orientation of the lacunae, any 2D measurements are unreliable and cutting planes have to be chosen with utmost care. Figure 3 illustrates the vastly different appearance of lacunae depending on the cutting plane.

#### Influence of Eliminating Border Regions of Lacunae

**Fig. 5:**
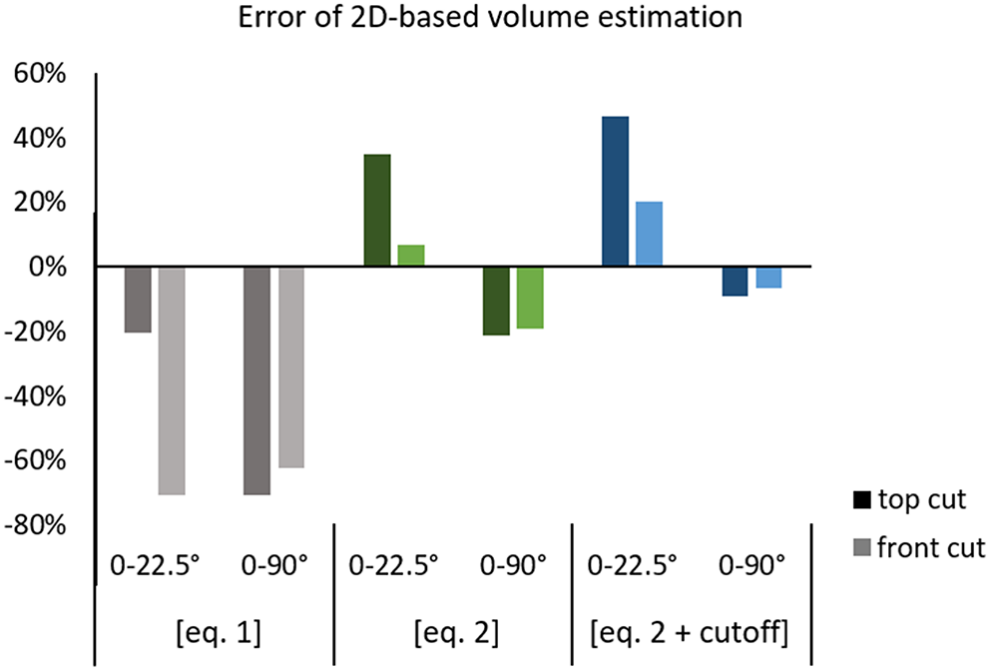
Errors of 2D-based volume estimation are reduced when using [Eq. 2]. The top cutting plane (approximately parallel to the longest axes of the artificial lacuna) mostly results in higher errors than the frontal cutting plane (approximately along the longest and shortest axis). Using a cutoff shifts the error by 3–7%

To limit the influence of very small lacunae cross-sections originating from lacunae cut close to their circumference, a minimum area cutoff can be used and only lacunae with an area of more than 15 μm² might be included in analyses [adapted for 2D from Hemmatian, et al. [46]]. But this only changes the error in estimation by 4–7%, see Fig. 5 and supplementary information for details.

### Suggestions for Improvement for 2D-Based Lacunar Volume Estimation

To counteract the systematic underestimation, we tested an adaptation to [Eq. 1], relying more strongly on the major diameter of the measured ellipsoid-shaped lacuna area:

2 $${Lc.V}_{new}=\frac{\pi}{6}*{D}_{\text{m}\text{i}\text{n}\_cut}*{D}_{\text{m}\text{a}\text{x}\_cut}*{D}_{\text{m}\text{a}\text{x}\_cut}$$

Using this equation will result in an overestimation for realistic cutting planes (parallel to longest axis), but this overestimation is lower than the previously common underestimation using [Eq. 1]. Assuming realistic orientations between 0 and 22.5° and the sections cut approximately parallel to the long axes of the lacunae, the overestimation will be between 7 and 35% for 0.2 μm resolution for planes aligned with the longest and shortest or medium axis respectively. While using [Eq. 2] results in an overestimation for lacunar orientations between 0-22.5° it results in an underestimation for random orientations but both deviations are smaller than those obtained by [Eq. 1]. Note that we excluded perfectly aligned lacunae from this comparison, as these do not constitute a realistic scenario. Further we excluded the cutting plane aligned with the short and medium axis as these are rarely used in practice and are not recommended since cutting along this plane always drastically underestimates the volume independent of the used equation.

Figure 5 compares errors of 2D-based lacunar volume estimations calculated with [Eq. 1], [Eq. 2]or [Eq. 2] with a lower cutoff analyzing only lacunar cross-sections with more than 15 μm². For this figure, realistic lacunar orientations of 0-22.5° (lamellar bone) and 0–90° (woven bone) and realistic cutting planes (frontal and top) parallel to the long lacuna axis are compared.

While employing [Eq. 2] leads to more realistic estimations of lacunar volume, the presented data clearly shows a strong influence of lacunar orientation and cutting planes. Thus, whenever possible 3D imaging of lacunae should be used when the goal is to assess lacunae morphology. If 2D-based estimations are performed, [Eq. 2] might provide more realistic estimations for lacunar volume. Using a cutoff to filter for lacunae with > 15 μm² area only offsets the results. Hence, it corrects volumes calculated with [Eq. 1] slightly but is only beneficial when using [Eq. 2] for randomly oriented lacunae.

### Limitations of 2D-3D Comparison

The investigated artificial lacunae were created as perfect ellipsoids. When cutting an ellipsoid (= lacuna), the resulting cross-section is a perfect ellipse. When evaluating these ellipses with Fiji Analyze Particles, the algorithm employs the fitting of an ellipse to the artificial lacuna cross-sections, leading to optimal estimation conditions. In reality, multiple factors will hamper both the measurement of cut ellipses and estimation of the original lacunar dimensions: (1) The lacuna shape may not represent a perfect ellipsoid and subsequently will not represent a perfect ellipse when cut. (2) The imaging methods might introduce artifacts. (3) The gray value segmentation will be influenced by the thresholding algorithm and partial volume effect depending on the resolution and the selected method. As these factors are not present in the performed idealized comparison, it is very likely that specifically due to the partial volume effect observed over- or underestimations are resolution dependent. The influence of the imaging method, resolution, and thresholding on the accuracy of lacunae morphology parameters should be investigated further. Consequently, for recommendations regarding the resolution we rely on the published resolution dependency by Mader et al. [81], ref. Sect. Considerations for Nominal Resolution/Voxel Size.

Further we did not determine the influence of different lacunar shapes or axes ratios on the over- or underestimation. For more elongated lacunae, the underestimation is likely even stronger, while for rounder lacunae the suggested adapted equation might not provide a better outcome. More research is needed to elucidate this issue. This underlines the need to acquire sufficient preliminary knowledge about the lacunar shape when chosing 2D analysis.

Further, while we chose lacunar volume as an example to highlight the limitations of 2D-based estimation of 3D parameters, similar issues with error-prone estimations would occur for other morphological investigations such as shape determination or, for higher resolution methods, canaliculi assessment.

## Method-Independent Practical Recommendations

Several prerequisites have to be fulfilled for reliable osteocyte/lacuna morphometry in 2D and 3D: (1) A reasonably sized, representative volume/region of interest with an appropriate sample size has to be chosen. (2) The chosen resolution should be good enough to reliably detect expected effect sizes. For 2D additional requirements are necessary: (3) The orientation of the lacunae should be known as reasoned in the Sect. Influence of Lacunar Orientation. (4) The method-inherent underestimation of lacunar volume has to be considered.

### Sample Size Considerations

To fulfill criteria 1, the following section contains considerations on how to determine the necessary number of lacunae per sample and number of samples per group.

#### What is an Appropriate Number of Lacunae per Sample and Sample Size in 2D?

If 3D analysis of lacunae is not an option, the researcher should analyze an appropriate number of lacunae to ensure reproducibility. Many publications rely on fixed measurement region sizes to ensure comparability of samples from different groups [96]. While this is in general feasible and great for intraindividual comparisons, it brings a possible caveat. If the representative region size is chosen based on one study group and bone type (woven vs. lamellar), it is possible that the lacunae density in one of the other study groups or bone types is much lower. Table 3 presents exemplary osteocyte and lacunar densities for different tissue types to illustrate the broad range of values.

**Table 3:** Exemplary overview of osteocyte and lacunar densities in different bone tissue types. 3D-based results formatted in bold

Bone tissue type	Osteocyte/lacunar number density
**Human**	
Adult – cortical lamellar bone	~ 500–900 lacunae/mm² [97] ~ 150–210 osteocytes/mm² [98] **~ 15,000–17,000 lacunae/mm**^**3**^ [99]
Children – cortical lamellar bone	~ 300–380 lacunae/mm² [100, 101]
Children – cortical woven bone (fetal)	~ 700 lacunae/mm² [101]
Children – cortical bone (OI Type V)	~ 820 lacunae/mm² [100]
**Rodent**	
Cortical lamellar bone	~ 830 lacunae/mm² [102] **~ 73,000 lacunae/mm³** [103]
Cortical woven bone (fracture callus)	~ 1880 lacunae/mm² [102] **~ 100,000 lacunae/mm³** [103]
Trabecular lamellar bone	~ 1190 lacunae/mm² [102]
Primary trabecular bone (growth plate)	~ 1670 lacunae/mm² [102]

If pre-characterization with 3D methods is unavailable for example for synchrotron experiments, the use of 2D methods to perform an estimation of lacuna density might be appropriate. Then, although the same region size is evaluated, the number of evaluated lacunae can vary substantially. Therefore, the representative region size should be chosen based on the study group with the lowest expected lacunar density and fixed at a volume size that includes an appropriate number of lacunae.

We aimed to determine how many lacunae have to be measured to achieve a reproducible lacunar volume with a small relative standard error. For this, we randomly selected cross-sectional areas of our artificial bone volume containing 500 identical lacunae (0-22.5° orientation) and calculated the lacunar volume using [Eq. 2].

**Fig. 6:**
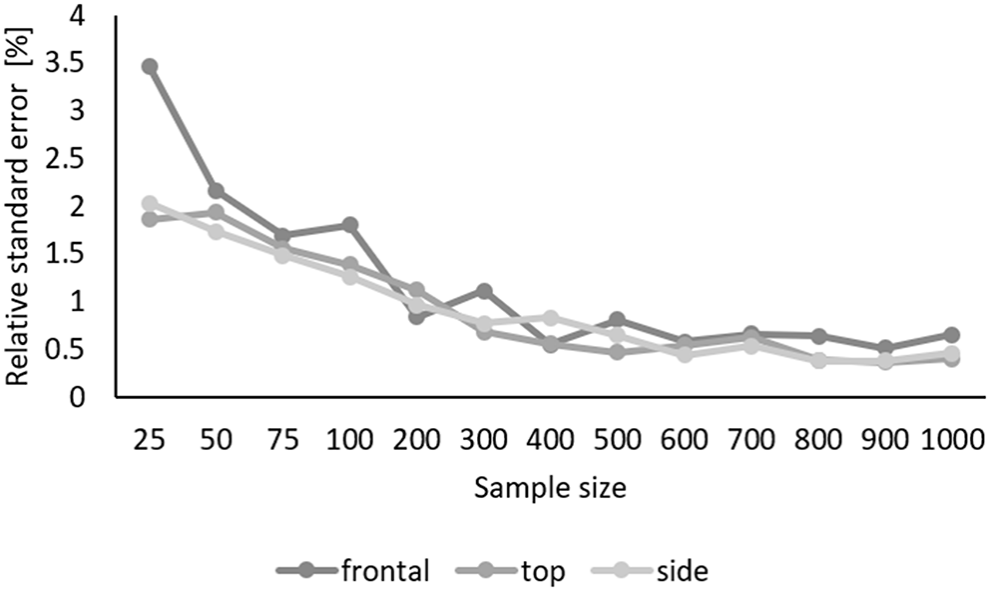
The relative standard error of mean lacunar volumes converges between 300 and 400 evaluated lacunae to less than 1% for all cutting planes. Top: cutting plane parallel to long and medium axis, frontal: cutting plane parallel to long and short axis, side: cutting plane parallel to medium and short axis

Then we repeatedly evaluated (20x) different amounts of lacunae cross-sections. (25-1000 lacunae) and determined mean values for lacunar volumes and the relative standard error (RSE) of means. We found that the means and RSEs for high and low resolutions converged equally and independently of the section plane between 300 and 400 evaluated lacunae, reaching an RSE of less than 1% for 400 evaluated lacuna cross-sections. Consequently, we recommend measuring at least 400 lacunae per sample for 2D-based estimations. Figure 6 shows the development of the RSE for sample sizes between 25 and 1000 lacunae.

#### What is an Appropriate Number of Lacunae per Sample and Sample Size in 3D?

In addition to finding the appropriate method and resolution, it is crucial to define (a) the necessary number of lacunae per sample and (b) the necessary number of samples per group. As exact values for these numbers depend on method-inherent factors concerning resolution and contrast, here we will briefly provide general recommendations.

To calculate the necessary number of lacunae per sample N_lac_, we suggest the following steps:

1. Determine parameters of interest and consider which parameter is most sensitive to 1 voxel variations. E.g. for one-dimensional parameters such as length a change by one voxel has a greater percental influence than for the lacunar volume.
2. Determine the standard deviation relative to the mean s_rel_ of the most sensitive parameter for your study. If no previous literature values exist, it may be necessary to image exemplary samples from each group to estimate the standard deviation. For this example, we chose a relative standard deviation based on Dong, et al. [10] of 0.37, where a resolution of 1.4 μm was used to image lacunae.
3. Decide which desired margin of error relative to the mean d_rel_ is needed for the mean value per sample. For this example, we chose 3% as this is a realistic expectation for the repeated measures error using microCT [80].
4. Decide on a necessary alpha for the study and calculate the Z-score corresponding to the desired level of confidence Z_α/2_. For this example, we chose α = 0.05, with Z_α/2_=1.96.
5. Calculate N_lac_ using the following equation (adjusted from Serdar, et al. [104]:

3 $${N_{lac}} = {Z_{\alpha /2}}\,* \,{s_{rel}}/{d_{rel}} = 1.96 \,*\, 0.37/0.03 = 584.4$$

The chosen example leads to a recommended number of at least 585 lacunae per sample. For lower relative standard deviations (e.g. 0.18 based on Yu, et al. [90], for pooled data of human lacunae measured at 0.03 μm) or higher accepted margins of error (e.g. 0.05), this number decreases to 49.8 lacunae per sample.

For the calculation of the necessary number of samples per group N_sample_, the reader is referred to Serdar et al. [104], where recommendations for sample size calculation are given with practical recommendations. For this calculation, G-Power, a free, user-friendly software, may be used. For clinicals research projects, the reader should take into consideration which effect size would have clinical implications [104].

### Considerations for Nominal Resolution/Voxel Size

The choice of imaging resolution is highly dependent on the research questions. For methods at the higher end of the resolution range, such as volume SEM, resolution is typically not a concern for visualization of the lacunae morphology. In lower resolution techniques, most prominently desktop microCT, studies have shown that assessing lacunar number, orientation and stretch becomes inaccurate upwards of voxel sizes from around 1.5–2 μm [79, 81]. Additionally, lacunar volume is underestimated above ~ 1 μm voxel size and should be interpreted with care [81]. Hence, we suggest using a minimum resolution of 1.4 μm, but ideally 1 μm to adequately assess lacunae morphology in 3D.

### Considerations for Segmentation

For both 2D and 3D imaging, choosing an appropriate segmentation threshold to separate bone from lacunae is a relevant factor that influences volume, dimension, and shape calculations.

In techniques, where bone is clearly distinguishable from the background and pixel values are reproducible between samples, (e.g. qBEI of homogeneously mineralized samples) one adequately selected threshold may be used for all samples [105]. In other cases, where partial volume effects are prominent (e.g. microCT) or the pixel values are arbitrary (e.g. confocal microscopy, Volume SEM), individual thresholds per sample should be determined. This can be done manually [106], but to maintain reproducibility, histogram-based algorithms are commonly used (e.g. Otsu, IsoData) [3, 8, 9, 12, 46, 107]. Histogram-based algorithms compensate for gray value differences e.g. originating from variations in mineralization or brightness. If the image contrast is not adequate for threshold-based segmentation, as it may be the case in some applications of electron microscopy or techniques producing multi-color images (e.g. brightfield microscopy), manual segmentation may be necessary. As this might result in unfeasible amounts of work in large datasets, the use of artificial intelligence for automation has become available in recent years [65, 76]. An example for a freely available tool to segment e.g. volume EM images containing cellular material (osteocytes) is the trainable Weka 2D/3D plug-in in FIJI [108].

Limited data regarding the detailed influence of various thresholding methods for different imaging methods exists. Hence, we would like to highlight the necessity of studies investigating the effects of different thresholding methods on the measurement of lacunar properties, ideally in comparison to a known ground truth. Subsequently, investigators should carefully consider and verify their choice of thresholding method. After binarization of cavities in the bone, it should be noted that the segmented instances may include noise or other voids such as canals or imaging artifacts. We therefore recommend filtering the individual segmented objects. The easiest approach is to filter excluding particles below a minimum and above a maximum volume [109]. As expected sizes for lacunae lie between 200 and 600 µm^3^ [5], our recommendations align with the proposal by Schemenz, et al. [103] for at least excluding particles smaller than 50µm^3^ (canalicular junctions) [110] and larger than 2000 µm^3^.

A more precise method, as proposed by Goff et al. [3], involves the manual classification of several segmented objects into lacunae or non-lacunae and determining appropriate filtering cutoffs for various parameters based on this ground truth. Subsequently, the binarized image can be filtered based on these cutoff values.

### Suggestion of Parameters for 3D

While a range of different parameters describing the size, shape, anisotropy and distribution of lacunae have been proposed since the emergence of 3D analyses, we suggest reporting of a set of the most commonly used, reliable parameters, mostly in accordance with those suggested by Goff et al. [3] in Table 3.

Various other parameters have been introduced to describe the osteocyte lacunar network in more detail, most notably by Mader et al. [81] and Weinkamer et al. [9] providing a range of metrics to describe the local environment of the lacunae in relation to their neighboring populations [81] and the network characteristics [9]. We suggest that these parameters are reported if relevant to a specific research question, as they might only be applicable to large populations of lacunae and also difficult to interpret.

**Table 4:** Overview of recommended parameters for 3D lacunae morphology

Parameter	Abbreviation	Description
**Individual lacunar parameters, calculated for each lacuna, averaged across the entire population**		
Lacunar volume	Lc.V	Volume of the lacuna, calculated based of a surface mesh derived from Lewiner marching cubes algorithm [3].
Lacunar stretch	Lc.St	Describes how stretched a lacuna is. A Lc.St of 0 describes a perfect sphere, while a Lc.St of 1 is an infinitely stretched object. Calculated from normalized axes of an ideal-fit ellipsoid based of the lacuna’s inertia [48]. Less sensitive to resolution than other parameters describing the anisotropy of the lacunae [81].
Lacunar oblateness	Lc.Ob	Describes how plate-like or rod-like a lacuna is. A Lc.Ob of -1 describes an infinitely prolate object (rod), while a Lc.Ob of 1 is a perfect disk. Calculation is based on the same principal as Lc.St [81].
Lacunar sphericity	Lc.Sr	Relates the lacunar surface, calculated from a surface mesh, to that of a sphere with the same volume. While surface measurements are highly dependent on image resolution and post-processing steps, sphericity can provide insights into lacunar surface topology in a normalized fashion [111].
Lacunar angle	Lc. θ	Angle between the longest axis of the lacuna’s ideal-fit ellipsoid and a vector of biological relevance, e.g. the longitudinal axis of a long bone. If the lacunae are strongly oblate, the angle of the shortest axis may instead be reported [81].
**Global lacunar parameters**		
Lacunar number density	Lc.N/BV	The number of lacunae (Lc.N) per unit of bone volume (BV)
Lacunar porosity	Lc.TV/BV	The total lacunar volume (Lc.TV) per unit of bone volume

While it is common to calculate mean values for each sample and then use these mean values to calculate a group mean and standard deviation, this approach has drawbacks. First, calculating a standard deviation of means might mask the true variability. Second, only looking at the mean values might prevent the detection of different osteocyte/lacunae subpopulations. Hannah et al. [112] showed the presence of a bimodal distribution of osteocyte lacunae volume in human femora. Thus, a thorough analysis of osteocyte/lacunae parameters should include visualizing and interpreting a histogram of the collected measurements.

## Conclusion

The vast improvement of existing imaging methods and the development of new methods has opened up new opportunities to gain insight into osteocyte lacunae morphology. First and foremost, researchers aiming to investigate osteocytes or their lacunae must choose a method and resolution appropriate to answer their research question. Based on the expected effect size and a consideration of clinical significance, appropriate numbers of osteocyte lacunae per sample and sample sizes per group need to be determined. If due to the nature of the research question or unavailability of 3D methods, a 2D approach is chosen, we urge researchers to carefully consider cutting planes, lacunar orientations and potential underestimations of lacunar volume. In some cases, a combination of 2D and 3D methods may be necessary for a thorough investigation. For 3D methods specifically, researchers should carefully consider appropriate segmentation and resolution and discuss which parameters are relevant for their topic.

While determining lacunae morphology remains challenging and each method presents its own challenges, the opportunities for imaging lacunae in large numbers, with a high level of detail or with functional staining will considerably advance the understanding of the role of osteocytes and their lacunae in physiological and pathological conditions.

### Electronic Supplementary Material

Below is the link to the electronic supplementary material.

Supplementary Material 1

## Data Availability

No datasets were generated or analysed during the current study.
