# Supplementary material for: 2D vs. 3D Evaluation of Osteocyte Lacunae - Methodological Approaches, Recommended Parameters, and Challenges: A Narrative Review by the European Calcified Tissue Society (ECTS)
Source: Curr Osteoporos Rep. 2024 Jul 9;22(4):396–415. doi: 10.1007/s11914-024-00877-z (PMC11324773; doi:10.1007/s11914-024-00877-z)
Supplement: Supplementary file 1 — Supplementary Material 1 [file 11914_2024_877_MOESM1_ESM.pdf]

Supplementary Methods to

“2D vs. 3D evaluation of osteocyte lacunae: Common methods, recommended parameters, and pitfalls”

For section 3. Systematic error with 2D-based estimates for 3D objects

To provide data-based recommendations for an appropriate volume size and resolution, we created artificial 3D datasets representing lacunae with different orientations at different resolutions. These 3D datasets were digitally cut into 2D slices to emulate 2D methods and evaluated with ImageJ particle analyzer.

For the dataset creation the python module PyEllipsoid was used [package created by Andrei Shkarin] running on Spyder within Anaconda3. 500 lacunae were distributed in a cube in a random manner without overlap or contact. All lacunae had an approximate axes ratio of 4:2:1, with an approximated long axis of 18.9µm, with small deviations due to voxel size.

Simulated lacunae dimensions:

Resolution [µm]	Long axis [vx]	Medium axis [vx]	Short axis [vx]	Axes ratio		
				Long axis	Medium axis	Short axis
0.20	95	47	24	3.96	1.96	1
0.50	38	19	9	3.8	1.9	1
0.99	19	9	5	3.8	1.8	1
1.45	13	6	3	4.3	2.0	1

Volumes were saved as a stack of bitmap files. Individual bitmap files represented 2D cross-sections of the artificial lacunae and were used for 2D analysis. Volumes were analyzed in three planes. The first plane is the top plane of the artificial volume and parallel to the longest and the medium axis for lacunae within the reference plane, the second is the frontal plane of the volume and parallel to the longest and shortest axis, and the third is the side plane and parallel to the medium and the short axis. 2D analysis of largest and smallest diameter of ellipsoids was done using ImageJ “Analyze particles” with size = 1-infinity and circularity = 0-1 and show ellipses.

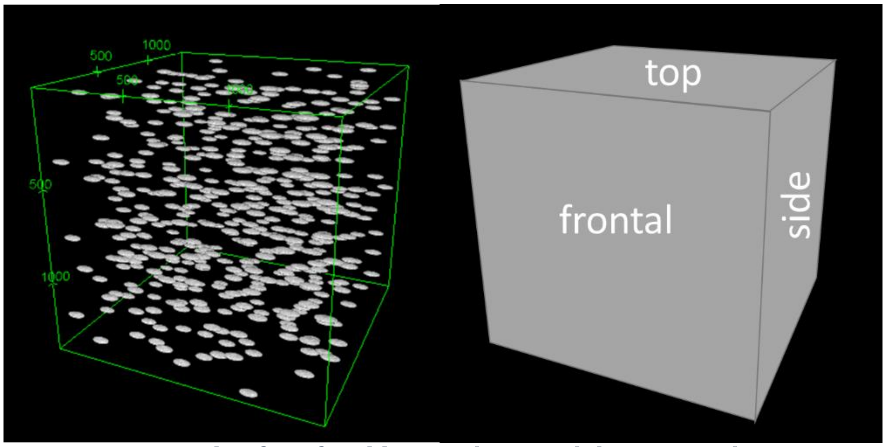

Figure S1: Example of artificial bone volume with lacunae with 0° orientation and cutting planes

For Section 3.1.2. Influence of lacunar orientation

The orientation of the lacunae was varied from parallel to reference axis (0°) to randomly angled to the reference plane in an angle between 0-22.5° and 0-90°. Lacunar volume estimations were performed for all three cutting planes.

Exported major and minor diameters from Fijis Analyze Particles were used to calculate the estimated 3D volume based on equations [1] and [2] using python.

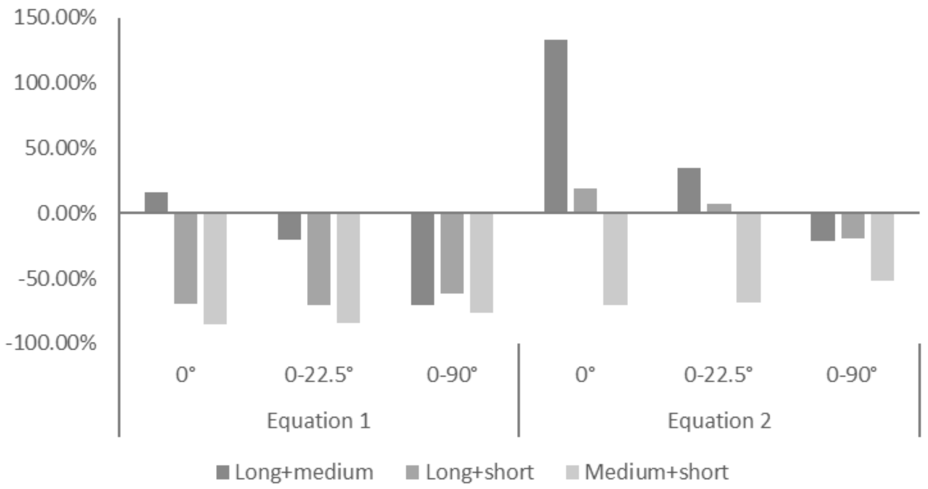

Figure S2: Error of 2D-based estimations of lacunar volume in dependence of lacunar orientation (0°, 0-22.5°, 0-90°) and cutting plane (parallel to long and medium axis (=top cut), to long and short axis (=front cut) or to medium and short axis (=side cut) (not recommended)).

To assess the influence of filtering small particles (=peripheral lacunar cross-sections), lacunar volume was calculated with equation [1] once for all lacunae cross-section and once only for lacunae with an area over 15µm².

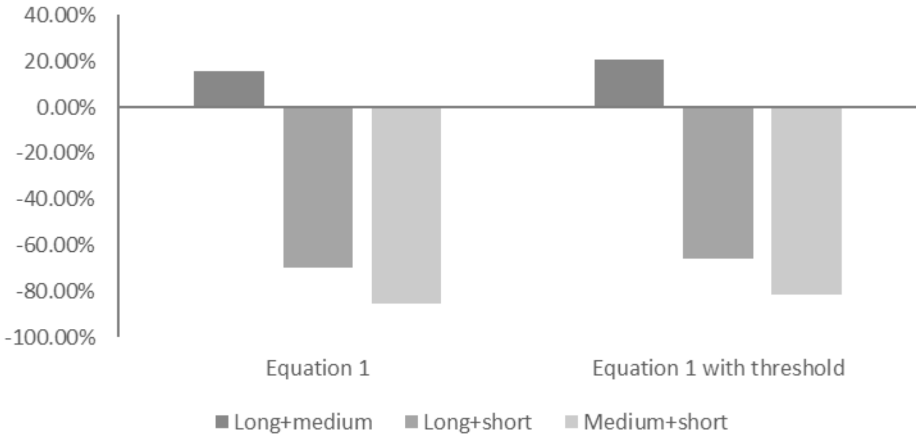

Figure S3: Error of 2D-based estimations of lacunar volume for equation 1 with or without a lacunar threshold of 15µm².

For section 4.1.1 What is an appropriate number of lacunae per sample and sample size in 2D?

To investigate how many lacunae have to be analyzed in 2D to reach a stable estimation, mean lacunar volumes were calculated for randomly picked 25, 50, 75, 100, 200, 300, 400, 500, 600, 700, 800, 900, and 1000 lacunar cross-sections. This calculation was repeated 20 times to determine the relative standard error for the mean values.
